# Exploring the cholesterol‐lowering effects of cereal bran cell wall‐enriched diets

**DOI:** 10.1002/fsn3.4141

**Published:** 2024-04-18

**Authors:** Tabussam Tufail, Farhan Saeed, Tanazzam Tufail, Huma Bader Ul Ain, Muzzamal Hussain, Sana Noreen, Mohd Asif Shah

**Affiliations:** ^1^ School of Food and Biological Engineering, Jiangsu University Zhenjiang China; ^2^ University Institute of Diet & Nutritional Sciences The University of Lahore Lahore Pakistan; ^3^ Faculty of Health and Life Sciences, Persiaran Perdana BBN, 71800 Putra Nilai, Nilai INTI International University Negeri Sembilan Malaysia; ^4^ Department of Food Sciences Government College University Faisalabad Faisalabad Pakistan; ^5^ Faisalabad Medical University Faisalabad Pakistan; ^6^ Department of Economics Kabridahar University Kabridahar Somali Ethiopia; ^7^ Centre of Research Impact and Outcome, Chitkara University Institute of Engineering and Technology Chitkara University Rajpura Punjab India; ^8^ Division of Research and Development Lovely Professional University Phagwara Punjab India

**Keywords:** cell wall, cereal grain, hypercholesterolemia, polysaccharides

## Abstract

Cell wall polysaccharides (dietary fiber) in cereal grains contribute to health benefits. The novelty of the current study was an effort to explore the in vivo therapeutic potential of different cereal bran cell walls against hypercholesterolemia. For this purpose, the cell walls were isolated from different cereal brans (wheat, maize, oats, and barley), and the intake of these cereal bran cell walls was evaluated for their anti‐lipidemic activity in normal and hypercholesterolemic rats. The serum taken from the rats was tested for cholesterol, lipid, and triglyceride profiles before and after treatment. The outcomes of the current study have shown that the cereal cell wall has a significant hypercholesterolemia effect. The biochemical parameters of the control animals were within the normal clinical ranges, indicating that the experimental diets were safe. Among cereal bran cell walls, barley bran significantly decreased cholesterol (56.35 ± 1.35 mg/dL), low‐density lipoprotein (56.35 ± 1.05 mg/dL), triglycerides (105.29 ± 1.95 mg/dL), and increased high‐density lipoprotein level (48.35 ± 1.35 mg/dL). These findings provide conclusive evidence that the cereal cell wall is beneficial in the treatment of hypercholesterolemia and may potentially provide protection against other acute, recurring, or chronic illnesses.

## Graphical abstract

The cereal cell wall may inhibit the activity of cholesterol acyltransferase, which acts in the re‐esterification of cholesterol in the small intestine. Overall, cell walls exhibited significant hypocholestrolemic activity, which suggests that the concentrations of cell walls obtained from cereals have many beneficial aspects along with hypocholestrolemia.

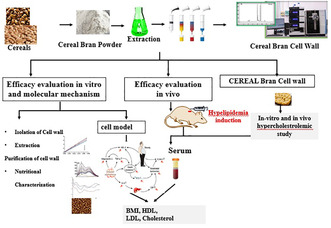

## 1 INTRODUCTION

Cereals, belonging to the Gramineae family, are extensively cultivated in temperate and tropical areas. They provide over fifty percent of the energy utilized in plant‐based diets, including but not limited to wheat, rice, and maize (Tufail et al., 2020). Europe's most widely cultivated cereal crop is wheat, followed by barley and oats in that order of prominence, respectively. Cereary grain comprises three primary constituents: embryo, endosperm, and bran, which is multilayered for reinforcement. The composition and properties of the cell wall of the complex structure that is the cereal kernel vary. The predominant constituent of the composition is starch, with proteins and non‐starch polysaccharides accounting for 10%–15%, 3%–8%, and 70%–80%, respectively, of the total. Dietary or pharmaceutical reduction of total and LDL lowers CHD risk. Whole‐grain diets reduce the risk of cancer, cardiovascular disease, and noninsulin‐dependent diabetes. Whole‐grain fiber affects risk factors like blood glucose, insulin, and cholesterol, which may explain these findings. Studies have shown that soluble‐fiber‐rich whole grains like oats and barley reduce blood cholesterol better than insoluble‐fiber‐rich grains like wheat or rice. Adding dietary fiber, a variety of plant compounds that resist digestion by human gastrointestinal enzymes, lowers cholesterol (Noreen, Tufail, Ul Ain, et al., 2023). The structural or matrix fibers (lignins, cellulose, and some hemicelluloses) are insoluble in water, while the natural gel‐forming fibers (pectins, gums, mucilages, and the rest of the hemicelluloses) are soluble. Qualitative reviews linked soluble fibers such as oats, psyllium, pectin, barley, and guar gum to reducing total and LDL‐C (Tufail, Saeed, Pasha, et al., 2021).

Bran is the hard outer layer of grain left over after milling. Thick‐walled cells in cereal bran include aleurone, testa, pericarp, and remnant endosperm tissue. Bran is found in rice, corn, wheat, oats, barley, rye, and millet (McRorie Jr et al., 2020; Semjon et al., 2020). Cereal bran is high in fiber, essential fatty acids, carbohydrates, protein, vitamins, and minerals (El Rabey et al., 2013; Sun et al., 2005). It also contains phytic acid, which inhibits nutritional absorption (Bader Ul Ain et al., 2019; Ciudad‐Mulero et al., 2020; Jefferson & Adolphus, 2019; McIntosh et al., 1991). Cereal bran is added to muffins and breakfast cereals to boost fiber consumption. Cereal bran can be pickled (nukazuke) like Japanese tsukemono. Cell wall composition and characteristics vary greatly (Hazen et al., 2003; Reddy et al., 2000; Revanappa et al., 2010). Starch, proteins, and non‐starch polysaccharides make up 70%–80%, 10%–15%, and 3%–8%, respectively. Different cell wall components have different structural and functional complexity (Awika et al., 2018; Balyan et al., 2013; Bondia‐Pons et al., 2013; Călinoiu & Vodnar, 2018; Rokhade & Rao, 2005). Research by Comino et al. (2014) found that β‐glucan has a weak cell wall, but arabinoxylan is well‐connected in rye, wheat, and hull‐less barley endosperm flours.

Merali et al. found that hydrothermal pretreatment opens wheat bran cell walls, breaks down, solubilizes, and hydrolyzes hemicellulosic arabinoxylans and glucose, and reduces cross‐linking phenolic acids (Merali et al., 2015; Tufail et al., 2018; Tufail, Saeed, Afzaal, et al., 2021). Dabour et al. (2022) also probed the effects of barley bran and oat bran dietary fibers on liver cholesterol range. This diet (including dietary cholesterol) decreased serum LDL, liver cholesterol, and liver lipid and raised HDL and HDL/LDL cholesterol ratios (Noreen, Tufail, Badar Ul Ain, et al., 2023). Hypocholesterolemia may be caused by increased bile acid excretion, fermentation‐produced short‐chain fatty acids, or small intestinal food absorption. In a crossover research, Whyte et al. explained how oat and wheat bran affect plasma lipids (Whyte et al., 1992). High‐soluble oat fiber diets can significantly cut plasma total and LDL cholesterol in mildly hypercholesterolemic patients, lowering coronary heart disease risk (Tufail, Saeed, Afzaal, et al., 2021). Considering all the above, the cereal bran cell wall must be isolated and purified and used in goods to fight various diseases. Soluble fiber has the ability to bind cholesterol in the bowel and eliminate it from the body. This is because soluble fiber is not absorbed in the intestine during digestion. Water, protein, fat, carbohydrates, arabinoxylans, cellulose, lignin, fructans, β‐glucans, nonstarch polysaccharides, phenolic acids, flavonoids, tocopherols, tocotrienols, c‐oryzanol, and phytic acid are present in the bran cell wall (Noreen et al., 2021). The current study extracted and purified wheat, rice, maize, barley, oat, rye, and oat bran cell walls for nutritional characterization, product integration, and hyperglycemia and hyperlipidemia prevention.

## 2 MATERIALS AND METHODS

### 2.1 Procurement of raw materials

Two varieties of four cereal brans, i.e., wheat, barley, maize, and oat, were procured from Faisalabad.

### 2.2 Isolation of cell walls

Cell walls from various grain brans were isolated and purified, and carbohydrates and proteins from endosperm tissue were extracted using the method of Brillouet et al. (1988). “First, 50 g bran was suspended in 0.1 M phosphate buffer (pH 7.5 I 1) with 1% sodium lauryl sulfate 2‐mercaptoethanol and 1% sodium azide. Add 25 ml of 5 mg/mL alkaline protease to buffer and stir for 2 h at 40°C. The residue was rinsed and suspended in distilled water following centrifugation. After boiling for 15 min, 10 mL Termamyl 120 L was added and stirred at 95°C for 1 h. A sintered glass filter collected the residue, which was rinsed with 500 mL hot water, solvent‐exchanged (ethanol, acetone), and oven‐dried overnight at 40°C”.

#### 2.2.1 Experimental animals

The hypocholestrolemic potential of wheat, maize, oats, and barley cell walls was tested on rats. The Animal Room of the Department of Physiology, Government College University, Faisalabad, housed 80 male Sprague Dawley rats (180–200 g). For 1 week, rats were fed a baseline diet to acclimate. Environmental conditions during the experiment included a 23 ± 2°C temperature, 55 ± 5% relative humidity, and a 12‐h light–dark cycle. Dissecting certain rats at trial start provided baseline values for specified attributes. The Research Ethical Committee of Government College University, Faisalabad, accepted the methods.

### 2.3 Diet

#### 2.3.1 Control diet

The control diet (T_0_) contained maize oil (10%), corn starch (66%), protein (10%), cellulose (10%), minerals (3%), and vitamin combination (1%) (Noreen, Rehman, et al., 2023).

#### 2.3.2 High cholesterol diet

Normal rats will be fed a high‐cholesterol diet of 2.0% cholesterol and 0.5% cholic acid to produce hypercholesterolemia in all groups except T_0_. To monitor hypercholesterolemia induction, rats were examined periodically (Feng et al., 2019; Noreen, Rehman, et al., 2023).

### 2.4 Kits

The Gamma Trade Company provided TC, LDL‐C, VLDL, and HDL for pharmaceutical and chemical purposes.

### 2.5 Animal experiment

After 1 week of adaptation, 80 rats were divided into two groups. As a negative control group (−ve), Group I (16 rats) was fed only the basal diet. Group II (64 rats) received a hypercholesterolemic diet without any medications for 2 weeks during the trial (Noreen, Rehman, et al., 2023). This group had four 16‐rat subgroups. Disease‐free male rats with an age range of 6–8 weeks and a weight of 180–200 g were included in the study, and diseased, underweight, and female rats were excluded from the study. Table 6 shows that the other four subgroups were fed 2% wheat, barley, maize, and oat bran cell walls for 3 weeks.

**TABLE 1:** Treatment plan.

Treatments	Addition of cell wall in diet (%)
T_0_	Control diet
T_1_	2% wheat bran cell wall
T_2_	2% barley bran cell wall
T_3_	2% maize bran cell wall
T_4_	2% oat bran cell wall

### 2.6 Biochemical examination

Hematological parameters were measured in EDTA‐treated tubes for baseline and follow‐up testing. Fortnightly serum lipid profiling included cholesterol, LDL, HDL, and TG. The total cholesterol was determined using the Desai et al. (2006) method. HDL was measured using the technique mentioned by Alshatwi et al. (2010). LDL levels in sera samples were also reported according to Kim et al. (2011). Triglycerides were measured using the methodology reported by Patel et al. (2013).

### 2.7 Statistical analysis

Following parameter data collection, Statistix 8.1 was utilized to analyze the statistics. In the preliminary analysis of variety composition, a complete randomized design (CRD) was utilized. Analysis of variance (two‐factor factorial) and least significant difference were subsequently utilized by Steel et al. to establish treatment significance over variety (Steel & Torrie, 1980).

## 3 RESULTS

### 3.1 Cell wall content of cereals

The highest cell wall contents (8.42 ± 0.11 g/100 g) were present in barley, followed by wheat (5.69 ± 0.10 g/100 g) and oat (4.22 ± 0.02 g/100 g), whereas maize showed the lowest cell wall contents (4.09 ± 0.11 g/100 g) (Table 2).

**TABLE 2:** Mean values for cell wall contents (g/100 g) of cereals bran.

Cereals	Wheat	Barley	Maize	Oat
Cell wall content	5.69 ± 0.10^b^	8.42 ± 0.11^c^	4.09 ± 0.11^a^	4.22 ± 0.02^a^

*Note*: Different letters in the table indicate statistically significant differences between the samples while the level of significant is *p*= < .05.

### 3.2 Efficacy studies in normal and hypercholesterolemic rats

#### 3.2.1 Total cholesterol (TC)

Cholesterol levels after consuming cereal grain cell walls are in Table 3. In the control group (T_0_), maximum cholesterol levels (62.30 ± 1.88 mg/dL and 62.84 ± 2.62 mg/dL) were observed in trial I and trial II of study II (anti‐hypercholesterolemic study). Normal rat cholesterol levels ranged from 83.07 ± 2.11 to 82.95 ± 1.03 mg/dL. Both tests showed the greatest cholesterol decrease in T_2_ (barley cell wall). T_2_ showed lower cholesterol levels of 77.22 ± 2.98, 76.16 ± 1.04, 56.70 ± 2.91, and 56.35 ± 1.35 mg/dL in trials I and II of normal and hypercholesterolemic investigations. T_3_ (maize cell wall) showed a considerable cholesterol drop in both experiments. T_3_ cholesterol levels decreased to 82.64 ± 5.28 mg/dL in the normal study and 81.32 ± 3.55 mg/dL in the hypercholesterolemic study, respectively. Trials I and II of the normal study and trial I and II of the hypercholesterolemic study showed lower cholesterol levels (80.19 ± 4.66, 79.25 ± 2.21, and 58.11 ± 3.11, correspondingly) in T_1_. In T_4_, cholesterol levels decreased to 81.44 ± 1.12, 80.82 ± 4.05, 59.29 ± 3.34, and 60.47 ± 2.96 mg/dL in normal and hypercholesterolemic studies, respectively.

**TABLE 3:** Effect of treatments on cholesterol level.

Treatments	T_0_	T_1_	T_2_	T_3_	T_4_
Study I					
Trial I	83.07 ± 2.11	80.19 ± 4.66	77.22 ± 2.98	82.64 ± 5.28	81.44 ± 1.12
Trial II	82.95 ± 1.03	79.25 ± 3.21	76.16 ± 1.04	81.32 ± 3.55	80.82 ± 4.05
Study II					
Trial I	62.30 ± 1.88	58.11 ± 3.11	56.70 ± 2.91	61.53 ± 1.01	59.29 ± 3.34
Trial II	62.84 ± 2.62	58.40 ± 4.53	56.35 ± 1.35	61.23 ± 1.66	60.47 ± 2.96

*Note*: T_0_ = control diet; T_1_ = wheat bran cell wall; T_2_ = barley bran cell wall, T_3_ = maize bran cell wall; T_4_ = oat bran cell wall.

#### 3.2.2 Low‐density lipoprotein (LDL)

Table 4 shows that wheat, barley, maize, and oat cell walls significantly lowered LDL in both experiments. The highest LDL level (33.18 ± 1.68 mg/dL) was seen in T_0_. The experimental diets T_1_ (30.19 ± 1.66 mg/dL), T_2_ (27.22 ± 0.98 mg/dL), T_3_ (32.64 ± 0.64 mg/dL), and T_4_ (31.44 ± 1.02 mg/dL) significantly reduced LDL. Similarly, in trial II, LDL levels decreased by 33.17 ± 4.03, 30.25 ± 3.01, 27.16 ± 304, 32.32 ± 3.25, and 31.82 ± 0.05 mg/dL for T_0_, T_1_, T_2_, T_3_, and T_4_, in normal study. In hypercholesterolemic study groups, the control group had the highest LDL level (62.10 ± 1.08 mg/dL), followed by maize bran cell wall fed rats (61.29 ± 0.41 mg/dL). In experiment I, rats fed barley bran cell walls had the lowest LDL values (56.35 ± 1.05 mg/dL). The highest LDL level (62.34 ± 2.62 mg/dL) was seen in T_0_ in trial II, with decreasing levels in T_3_, T_4_, T_1_, and T_2_ (56.35 ± 1.05 mg/dL).

**TABLE 4:** Effect of treatments on low‐density lipoprotein level.

Trials	T_0_	T_1_	T_2_	T_3_	T_4_
Study I					
Trial I	33.18 ± 2.01	30.19 ± 1.66	27.22 ± 0.98	32.64 ± 0.27	31.44 ± 1.02
Trial II	33.17 ± 4.03	30.25 ± 3.01	27.16 ± 3.04	32.32 ± 3.25	31.82 ± 0.05
Study II					
Trial I	62.10 ± 1.08	58.11 ± 0.11	56.70 ± 2.01	61.53 ± 0.41	59.29 ± 3.04
Trial II	62.34 ± 2.62	58.40 ± 1.53	56.35 ± 1.05	61.23 ± 1.16	60.47 ± 1.96

*Note*: T_0_ = control diet; T_1_ = wheat bran cell wall; T_2_ = barley bran cell wall, T_3_ = maize bran cell wall; T_4_ = oat bran cell wall.

#### 3.2.3 High‐density lipoprotein (HDL)

The highest HDL level (48.70 ± 1.35 mg/dL) was obtained in trial II (T_2_), whereas the lowest was observed in trial I (T_0_) (Table 5). In trial I, T_2_ had the highest HDL level (42.22 ± 1.99 mg/dL), followed by T_1_ (39.19 ± 1.16 mg/dL), T_4_ (38.44 ± 3.12 mg/dL), T_3_ (37.64 ± 1.28 mg/dL), and T_0_ (36.27 ± 0.41 mg/dL). In trial II, T_2_ had the highest HDL level (43.16 ± 1.14 mg/dL), followed by T_1_ (39.25 ± 0.21 mg/dL), and T_4_ (38.82). T_2_ (barley bran cell wall) had the highest HDL (48.35 ± 0.91 mg/dL), while T_0_ (control/basal diet) had the lowest (42.11 ± 1.08 mg/dL) during trial I. In trial II of the anti‐hypercholesterolemic investigation, HDL values for T_0_, T_1_, T_2_, T_3_, and T_4_ were 42.04 ± 0.42, 46.40 ± 0.53, 48.70 ± 1.35, 43.23 ± 2.16, and 45.47 ± 2.61 mg/dL, respectively. Trials I and II rats administered barley bran cell wall had the highest percentage.

**TABLE 5:** Effect of treatments on high‐density lipoprotein level.

Trials	T_0_	T_1_	T_2_	T_3_	T_4_
Study I					
Trial I	36.27 ± 0.41	39.19 ± 1.16	42.22 ± 1.99	37.64 ± 1.28	38.44 ± 3.12
Trial II	36.52 ± 2.03	39.25 ± 0.21	43.16 ± 1.14	37.32 ± 0.55	38.82 ± 2.15
Study II					
Trial I	42.11 ± 1.08	46.11 ± 2.21	48.70 ± 0.91	43.53 ± 2.01	45.29 ± 0.34
Trial II	42.04 ± 0.42	46.40 ± 0.53	48.35 ± 1.35	43.23 ± 2.16	45.47 ± 2.16

*Note*: T_0_ = control diet; T_1_ = wheat bran cell wall; T_2_ = barley bran cell wall, T_3_ = maize bran cell wall; T_4_ = oat bran cell wall.

#### 3.2.4 Triglycerides (TG)

Rats given wheat, barley, maize, and oat bran cell walls had lower triglycerides (Table 6). In trial I of the normal study, triglycerides were measured at 63.13 ± 0.11, 60.79 ± 1.66, 56.29 ± 0.98, 62.44 ± 1.28, and 61.29 ± 2.12 mg/dL for T_0_, T_1_, T_2_, T_3_, and T_4_. Triglyceride levels in serum were observed as 62.05 ± 2.03, 58.15 ± 3.11, 55.35 ± 1.54, 61.29 ± 3.05, and 60.38 ± 0.05 mg/dL for T_0_, T_1_, T_2_, T_3_, and T_4_ in trial II of the normal investigation. In the antihypercholesterolemic investigation, T_0_ (control diet) had the highest triglycerides (114.10 ± 1.18 mg/dL), whereas T_2_ had the lowest (106.58 ± 4.91 mg/dL) during trial I. Similarly, in trial II, T_0_ had the highest triglyceride level (113.54 ± 2.52 mg/dL), followed by T_3_, T_4_, T_1_, and T_2_ (111.34 ± 1.16, 111.48 ± 2.46, 110.10 ± 4.13, and 105.29 ± 1.95 mg/dL, respectively).

**TABLE 6:** Effect of treatments on triglyceride level.

Trials	T_0_	T_1_	T_2_	T_3_	T_4_
Study I					
Trial I	63.13 ± 0.11	60.79 ± 1.66	56.29 ± 0.98	62.44 ± 1.28	61.29 ± 2.12
Trial II	62.05 ± 2.03	58.15 ± 3.11	55.35 ± 1.54	61.29 ± 3.05	60.38 ± 0.05
Study II					
Trial I	114.10 ± 1.18	111.45 ± 4.11	106.58 ± 4.91	113.78 ± 2.01	112.45 ± 1.34
Trial II	113.54 ± 2.52	110.10 ± 4.13	105.29 ± 1.95	112.34 ± 1.16	111.48 ± 2.46

*Note*: T_0_ = control diet; T_1_ = wheat bran cell wall; T_2_ = barley bran cell wall, T_3_ = maize bran cell wall; T_4_ = oat bran cell wall.

## 4 DISCUSSION

Thicker walled cereal bran cells contain aleurone, testa, pericarp, and endosperm tissue. Fiber, vital fatty acids, carbs, protein, vitamins, and minerals are abundant in cereal bran (El Rabey et al., 2013; Jefferson & Adolphus, 2019; Sun et al., 2005). Cereal bran reduces the risk of chronic diseases (Knudsen et al., 1995; Saeed et al., 2021). The findings of this research suggested that the mean levels of cholesterol were higher after consuming selected cereal grain cell walls, as shown in Table 1. The control group (T_0_) exhibited maximal cholesterol levels of 62.30 ± 1.88 mg/dL and 62.84 ± 2.62 mg/dL, respectively, in trials I and II of study II (anti‐hypercholesterolemic study). The mean cholesterol levels influenced by the cell walls of particular cereal grains were displayed. Maximum cholesterol levels for the control group (T_0_) were recorded as 62.30 ± 1.88 mg/dL and 62.84 ± 2.62 mg/dL, respectively, in both trials of study II (anti‐hypercholesterolemic study). In the normal study, T_3_ cholesterol levels were recorded as 82.64 ± 5.28 mg/dL, whereas in the hypercholesterolemic study, they were reduced to 81.32 ± 3.55 mg/dL. Trials I and II of the control group and trial I and II of the hypercholesterolemic group, respectively, observed reduced cholesterol levels in T_1_ (80.19 ± 4.66, 79.25 ± 2.21, and 58.11 ± 3.11, respectively). In normal and hypercholesterolemic studies, T_4_ cholesterol levels decreased to 81.44 ± 1.12, 80.82 ± 4.05, 59.29 ± 3.34, and 60.47 ± 2.96 mg/dL, respectively. The findings of this investigation mirrored those of a prior study conducted by AbuMweis et al., which explored the lipid‐lowering effects of barley beta‐glucan. In a dose‐dependent manner, barley beta‐glucan reduced total cholesterol and LDL (AbuMweis et al., 2010). Further, Moreira‐Rosario et al. discovered that wheat germ‐enriched bread did not change cardiovascular disease risk factors like cholesterol levels (Moreira‐Rosário et al., 2019). In a randomized controlled experiment, Momenizadeh et al. studied how oat and wheat bread affected hypercholesterolemic individuals' lipid profiles. Beta glucan in oat bread lowered cholesterol more than wheat bread (Momenizadeh et al., 2014).

According to Shimizu et al. (2019), barley supplementation also influenced lipid parameters and biomarkers associated with cardiovascular disease, including total cholesterol levels. Cha et al. also found that maize may also help in reducing cholesterol levels in rats. Oats decrease cholesterol due to beta‐glucan (Cha et al., 2016). Increased intestinal viscosity, delayed gastric emptiness, reduced intestinal mixing, entrapment of mixed micelles, restriction of nutrients, bile salts, and digestive enzymes mixing and transport are involved (Grundy et al., 2018; Gunness et al., 2016; Gunness & Gidley, 2010; Lairon et al., 2007; Mackie et al., 2016; Mälkki & Virtanen, 2001). Wheat, barley, maize, and oat cell walls significantly reduced low‐density lipoprotein (LDL) levels in both trials (Table 3). LDL levels peaked at 33.18 ± 1.68 mg/dL in T_0_. Experimental diets T_1_ (30.19 ± 1.66 mg/dL), T_2_ (27.22 ± 0.98 mg/dL), T_3_ (32.64 ± 0.64 mg/dL), and T_4_ (31.44 ± 1.02 mg/dL) significantly reduced LDL. Similarly, in trial II, LDL levels decreased by 33.17 ± 4.03, 30.25 ± 3.01, 27.16 ± 304, 32.32 ± 3.25, and 31.82 ± 0.05 mg/dL for T_0_, T_1_, T_2_, T_3_, and T_4_, in the normal study. In hypercholesterolemic study groups, the control group had the highest LDL levels (62.10 ± 1.08 mg/dL), followed by maize bran cell wall‐fed rats (61.29 ± 0.41 mg/dL) and barley bran cell wall‐fed rats (56.35 ± 1.05 mg/dL) in trial I. In trial II, the greatest LDL level (62.34 ± 2.62 mg/dL) was observed in T_0_, progressively decreasing in T_3_, T_4_, T_1_, and T_2_ (56.35 ± 1.05 mg/dL).

Mumford et al. showed that premenopausal women lacking estrogen had lower lipoprotein cholesterol after eating fiber (Mumford et al., 2011). Barley supplementation also altered cardiovascular disease biomarkers and lipid parameters, according to Behall et al. Because barley contains soluble fiber, total cholesterol dropped and high‐density lipoprotein increased (Behall et al., 2004). In a randomized controlled experiment, Tighe et al. (2013) found that wheat and oat‐based whole grain meals did not affect blood lipoprotein size and distribution in overweight middle‐aged people.

The maximum HDL level (48.70 ± 1.35 mg/dL) was obtained in trial II (T_2_), whereas the lowest was observed in trial I (T_0_) (Table 3). In trial I, T_2_ had the highest HDL level, followed by T_1_, T_4_, T_3_, and T_0_. In trial II, T_2_ had the highest HDL level, followed by T_1_ and T_4_. T_2_ (barley bran cell wall) had the highest HDL (48.35 ± 0.91 mg/dL), while T_0_ (control/basal diet) had the lowest value during trial I. In trial II of the anti‐hypercholesterolemic investigation, HDL values for T_0_, T_1_, T_2_, T_3_, and T_4_ were 42.04 ± 0.42, 46.40 ± 0.53, 48.70 ± 1.35, 43.23 ± 2.16, and 45.47 ± 2.61 mg/dL, respectively. In trials I and II, rats given barley bran cell walls had the highest percent growth. A study by Lupton et al. indicated that barley bran flour and oil raised total cholesterol in hypercholesterolemia individuals (Lupton et al., 1994). HDL increased dramatically, while total cholesterol, LDL, and triglycerides decreased. In a randomized controlled clinical trial, Gong et al. (2018) found that barley's high soluble fiber and beta glucan content reduced cholesterol better than wheat in hypercholesterolemic patients.

In rats, wheat, barley, maize, and oat bran cell walls significantly reduced triglycerides (Table 5). In trial I of the normal study, TG was measured at 63.13 ± 0.11, 60.79 ± 1.66, 56.29 ± 0.98, 62.44 ± 1.28, and 61.29 ± 2.12 mg/dL for T_0_, T_1_, T_2_, T_3_, and T_4_ Triglyceride levels in serum were observed as 62.05 ± 2.03, 58.15 ± 3.11, 55.35 ± 1.54, 61.29 ± 3.05, and 60.38 ± 0.05 mg/dL for T_0_, T_1_, T_2_, T_3_, and T_4_ in trial II of the normal investigation. In the antihypercholesterolemic investigation, T0 (control diet) had the highest triglycerides (114.10 ± 1.18 mg/dL), whereas T_2_ had the lowest (106.58 ± 4.91 mg/dL) during trial I. Similarly, in trial II, T_0_ had the highest triglycerides level (113.54 ± 2.52 mg/dL), followed by T_3_, T_4_, T_1_, and T_2_ (111.34 ± 1.16, 111.48 ± 2.46, 110.10 ± 4.13, and 105.29 ± 1.95 mg/dL, respectively).

Due to its ability to bind bile acids, soluble fiber derived from barley reduces total cholesterol, triglycerides, and LDL (Zhu et al., 2015). Jenkins et al. investigated the impact of wheat bran supplementation on the serum lipid profile and discovered that it significantly decreased the triglyceride concentration in hyperlipidemic individuals (Jenkins et al., 1999). Furthermore, the effect of oat bran on lipoprotein parameters was assessed in a placebo‐controlled study on subjects with hyperlipidemia, and it was observed that oat bran lowers the triglyceride level of lipid profile owing to the presence of high fiber contents (Bremer et al., 1991). Moreover, barley supplementation altered cardiovascular disease biomarkers and lipid parameters, according to Behall et al. (2004).

## 5 CONCLUSIONS

These findings suggest that the cell wall has direct effects on the absorption of cholesterol in the small intestine. In the small intestine, cholesterol re‐esterification is mediated by cholesterol acyltransferase, which may be inhibited by the cell wall. Among cereal bran cell walls, barley bran significantly decreased cholesterol (56.35 ± 1.35 mg/dL), low‐density lipoprotein (56.35 ± 1.05 mg/dL), triglycerides (105.29 ± 1.95 mg/dL), and increased high‐density lipoprotein level (48.35 ± 1.35 mg/dL). As a result, the cereal bran cell walls and barley bran were proposed as a novel option for the clinical care of hyperlipidemic patients. The cell wall exhibited substantial hypocholestrolemic activity, indicating that the concentrations of the cell wall should be investigated further.

## AUTHOR CONTRIBUTIONS

**Tabussam Tufail:** Formal analysis (equal); writing – original draft (equal). **Farhan Saeed:** Supervision (equal). **Tanazzam Tufail:** Formal analysis (equal); investigation (equal). **Huma Bader Ul Ain:** Writing – review and editing (equal). **Sana Noreen:** Software (equal). **Muzzamal Hussain:** Methodology (equal). **Mohd Asif Shah:** Data curation (equal).

## FUNDING INFORMATION

The authors received no financial support for the research, authorship, and/or publication of this article.

## CONFLICT OF INTEREST STATEMENT

All the authors declare that they have no conflict of interest.

## ETHICAL APPROVAL

The study was approved by the relevant ethical committee of Government College University, Faisalabad, with ethical No: GCUF/FS/20/18654.

## Data Availability

The data that were utilized for this analysis can be obtained upon request by contacting the respective author, even though all of the pertinent data have been supplied here.
